# Renal Function at Hospital Admission and Mortality Due to Acute Kidney Injury after Myocardial Infarction

**DOI:** 10.1371/journal.pone.0035496

**Published:** 2012-04-23

**Authors:** Rosana G. Bruetto, Fernando B. Rodrigues, Ulysses S. Torres, Ana P. Otaviano, Dirce M. T. Zanetta, Emmanuel A. Burdmann

**Affiliations:** 1 Division of Nephrology, Hospital de Base, Sao Jose do Rio Preto Medical School (FAMERP), Sao Jose do Rio Preto, São Paulo, Brazil; 2 Department of Internal Medicine - Division of Emergency and Chest Pain Center, Hospital de Base, Sao Jose do Rio Preto Medical School (FAMERP), Sao Jose do Rio Preto, São Paulo, Brazil; 3 Division of Cardiology, Hospital de Base, Sao Jose do Rio Preto Medical School (FAMERP), Sao Jose do Rio Preto, São Paulo, Brazil; 4 Public Health School, University of São Paulo, São Paulo, Brazil; University of Pittsburgh Medical Center, United States of America

## Abstract

**Background:**

The role of an impaired estimated glomerular filtration rate (eGFR) at hospital admission in the outcome of acute kidney injury (AKI) after acute myocardial infarction (AMI) has been underreported. The aim of this study was to assess the influence of an admission eGFR<60 mL/min/1.73 m^2^ on the incidence and early and late mortality of AMI-associated AKI.

**Methods:**

A prospective study of 828 AMI patients was performed. AKI was defined as a serum creatinine increase of ≥50% from the time of admission (RIFLE criteria) in the first 7 days of hospitalization. Patients were divided into subgroups according to their eGFR upon hospital admission (MDRD formula, mL/min/1.73 m^2^) and the development of AKI: eGFR≥60 without AKI, eGFR<60 without AKI, eGFR≥60 with AKI and eGFR<60 with AKI.

**Results:**

Overall, 14.6% of the patients in this study developed AKI. The admission eGFR had no impact on the incidence of AKI. However, the admission eGFR was associated with the outcome of AMI-associated AKI. The adjusted hazard ratios (AHR, Cox multivariate analysis) for 30-day mortality were 2.00 (95% CI 1.11–3.61) for eGFR<60 without AKI, 4.76 (95% CI 2.45–9.26) for eGFR≥60 with AKI and 6.27 (95% CI 3.20–12.29) for eGFR<60 with AKI. Only an admission eGFR of <60 with AKI was significantly associated with a 30-day to 1-year mortality hazard (AHR 3.05, 95% CI 1.50–6.19).

**Conclusions:**

AKI development was associated with an increased early mortality hazard in AMI patients with either preserved or impaired admission eGFR. Only the association of impaired admission eGFR and AKI was associated with an increased hazard for late mortality among these patients.

## Introduction

Recent North American and European epidemiological studies have shown that the incidence of acute kidney injury (AKI) is increasing at an alarming rate [1], [2]. The development and validation of the new AKI diagnostic criteria, namely RIFLE (Risk, Injury, Failure, Loss, and End-Stage Kidney Disease) [3] and AKIN (Acute Kidney Injury Network) [4], were significant advancements in the study of the AKI syndrome and permit accurate comparisons among different studies. The RIFLE criteria [3] classify AKI into increasing levels of severity. The first level, Risk, is defined as an abrupt (within 1–7 days) and sustained (>24 h) serum creatinine (SCr) increase of at least 1.5 from the reference SCr or a greater than 25% decrease in the glomerular filtration rate (GFR) compared to the reference GFR or a urine output of less than 0.5 mL/kg/h for more than 6 h.

Ischemic heart disease is the leading cause of death among adults in high-income countries and accounts for a substantial fraction of the total disease burden globally. AKI is an important and common complication after acute myocardial infarction (AMI), affecting from 10 to 55% of the patients (this latter number referring to patients suffering from cardiogenic shock) [5]–[7]. The development of AKI is associated with unfavorable outcomes and higher mortality after an AMI [5]–[10]. The mechanisms causing AKI in the first few days after an AMI are multifactorial, including systemic and renal hemodynamic changes secondary to an impaired cardiac output and an imbalance of vasodilators and vasoconstrictors, the use of contrast media, and immunological and inflammatory kidney damage resulting from crosstalk between the heart and the kidney [11].

The effect of pre-existing renal dysfunction on AKI mortality remains controversial and conflicting results have been published [12]–[16]. Similarly, very few studies have assessed the role of impaired estimated glomerular filtration rates (eGFRs) at hospital admission on the mortality of patients with AMI-associated AKI, and no studies have been explicitly designed to assess whether an impaired admission eGFR affects the prognosis of AMI-induced AKI as defined by the RIFLE criteria.

The purpose of this study was to evaluate the association of a decreased admission eGFR with the incidence and early and late mortality of patients developing AKI, as defined by the RIFLE criteria, in the acute phase of a myocardial infarction.

## Results

### Population characteristics

We evaluated 828 patients with a median age of 65 years (interquartile range: 54 to 74), 65.5% of whom were male. Our study population was composed of 7.7% black patients and 92.3% non-black patients. Overall, at the time of the study, 69% of the patients had a history of hypertension, 36.7% smoked, 25.7% were diabetic, 22.6% were dyslipidemic, 41.7% had previously used angiotensin-converting enzyme inhibitors (ACEIs)/angiotensin II receptor blockers (ARBs), 8.8% had prior percutaneous coronary intervention (PCI), 15.5% had been affected by prior coronary artery disease (CAD) with greater than 50% stenosis, 16.3% had suffered from prior infarction and 8.5% of the patients had previously undergone coronary artery bypass graft (CABG) surgery. The median SCr of patients upon hospital admission was 1.2 mg/dl (interquartile range: 1.0–1.5 mg/dL), and the admission median eGFR was 63.1 mL/min/1.73 m^2^ (interquartile range: 47.5–81.3 mL/min/1.73 m^2^).

The systolic left ventricular function (LVF) was measured in 709 patients and was classified as normal in 29.7%, mildly dysfunctional in 28.6%, moderately dysfunctional in 22.1% and severely dysfunctional in 19.6% of them. ST elevation myocardial infarction (STEMI) was diagnosed in 50.2% of the patients, 20.9% of whom presented with a Killip class >I and 54.5% of whom presented with an anterior wall infarction. The overall in-hospital mortality was 13.6%.

### AKI incidence and mortality

AKI occurred in 121 (14.6%) of the patients, with 76 patients of these patients (9.2%) in the Risk category, 37 patients (4.5%) in the Injury category and 8 patients (1%) in the Failure category. The 30-day mortality rate was 38.8% (8.8% in the group without AKI, p<0.001), and the 30-day to 1-year mortality rate was 25.4% (13% in the group without AKI, p-value = 0.006).

### Comparison between patients with impaired and non-impaired admission eGFRs

Patients with an impaired eGFR had a median admission SCr of 1.5 mg/dl (interquartile range: 1.3–1.8 mg/dL) and a median admission eGFR of 46.1 mL/min/1.73 m^2^ (interquartile range: 36.7–52.8 mL/min/1.73 m^2^). Patients with non-impaired eGFR had a median admission SCr of 1.0 mg/dL (interquartile range: 0.8–1.2 mg/dl) and a median admission eGFR of 79.2 mL/min/1.73 m^2^ (interquartile range: 68.5–96.1 mL/min/1.73 m^2^).

Among the study population, 46% had an admission eGFR<60 mL/min/1.73 m^2^. When compared to the group of patients with an admission eGFR≥60 mL/min/1.73 m^2^, this group was composed of a smaller proportion of men; was significantly older; and had a significantly increased prevalence of hypertension, diabetes, prior infarction, prior use of ACEIs/ARBs and previously documented CAD, PCI or CABG. A significantly higher number of patients with an admission eGFR≥60 mL/min/1.73 m^2^ had an admission heart rate (HR) >100 beats/min and a systolic blood pressure (SBP) <100 mmHg. These patients were also more likely to have non-ST elevation myocardial infarction (NSTEMI). When presenting with STEMI, these patients exhibited a higher frequency of Killip class >I (Table 1).

**Table 1:** Comparison of admission characteristics upon hospitalization between patients with not-impaired or impaired admission eGFR.

Characteristics	Total Cohort (n = 828)	a-eGFR≥60 (n = 447)	a-eGFR<60 (n = 381)	p-value**
Age (y)	65 (54–74)	59 (49–71)	70 (61–75)	<0.001
Male	65.5%	72.0%	57.7%	<0.001
History of hypertension	69%	57.9%	81.9%	<0.001
Current smoker	36.7%	44.5%	27.6%	<0.001
History of diabetes	25.7%	19.2%	33.3%	<0.001
Previous PCI	8.8%	6.7%	11.3%	0.021
Previous CABG	8.5%	5.1%	12.3%	<0.001
Prior CAD (stenosis >50%)	15.5%	10.7%	21.0%	<0.001
Previous AMI	16.3%	12.3%	21.0%	0.001
Prior use of ACEI/ARB*	41.7%	31.1%	54.1%	<0.001
STEMI	50.2%	55.9%	43.6%	<0.001
aSBP<100 mm Hg†	6.3%	3.6%	9.5%	0.001
aHR (beats/min)	80 (70–95)	80 (70–92)	80 (70–100)	0.249
aHR >100 beats/min	16.5%	13.2%	20.5%	0.005
Killip class >1 ‡	20.9%	15.6%	28.9%	0.001

a-eGFR, admission estimated glomerular filtration rate (mL/min/1.73 m^2^); PCI, percutaneous coronary intervention; CABG, coronary artery bypass graft; CAD, coronary artery disease; AMI, acute myocardial infarction; ACEI, angiotensin-converting enzyme inhibitors; ARB, angiotensin II receptor blockers; STEMI, ST elevation myocardial infarction; aSBP, admission systolic blood pressure; aHR, admission heart rate. Continuous variables are presented as median values (with interquartile ranges). Categorical variables are presented as percentages.

* n = 821 for the total cohort, n = 444 for a-eGFR≥60 mL/min/1.73 m^2^, and n = 377 for a-eGFR<60 mL/min/1.73 m^2^.

† n = 827 for the total cohort; n = 380 for a-eGFR<60 mL/min/1.73 m^2^.

‡ n = 416 (STEMI patients).

** comparison between eGFR≥60 and <60 mL/min/1.73 m^2^.

Patients with an impaired admission eGFR were significantly less likely to receive β-blockers, ACEIs/ARBs, and clopidogrel and were more likely to receive diuretics. Additionally, they were significantly less likely to undergo coronary angiography, PCI, CABG or any revascularization interventions during their hospitalization than those with an admission eGFR≥60 mL/min/1.73 m^2^. STEMI patients with an impaired admission eGFR were significantly less likely to receive thrombolytic treatment or any type of reperfusion therapy than subjects with an admission eGFR≥60 mL/min/1.73 m^2^. There was no difference between the two groups in the thrombolysis in myocardial infarction (TIMI) 3 flow rate or in the favorable reperfusion criteria after reperfusion treatment (table 2).

**Table 2:** Comparison between patients with not impaired and impaired admission eGFR regarding treatment, incidence of AKI, length of hospitalization and mortality.

Variables	Total Cohort (n = 828)	a-eGFR≥60 (n = 447)	a-eGFR<60 (n = 381)	p-value
ß-blockers	93.5%	95.1%	91.6%	0.043
ACEI/ARB	97.2%	98.7%	95.5%	0.006
Diuretics	57.2%	48.5%	67.5%	<0.001
Clopidogrel	82.2%	85.7%	78.2%	0.005
Coronary angiography	82%	89.3%	73.5%	<0.001
PCI	49%	53.7%	43.6%	0.004
CABG	6.4%	8.5%	3.9%	0.007
Any revascularization*	55.2%	61.5%	47.8%	<0.001
Thrombolytic treatment	33.6%	40%	23%	<0.001
Primary PCI for STEMI	48.3%	47.6%	47.6%	0.998
Any reperfusion therapy†	81%	86.4%	69.9%	<0.001
TIMI 3 flow rate after reperfusion treatment	86.5%	83.7%	90.8%	0.131
Favorable reperfusion criteria	73%	76.5%	66.9%	0.064
Incidence of AKI	14.6%	13.4%	16.0%	0.293
Length of hospitalization (d)	7.43 (4.4–13.3)	6.9(4.3–12.2)	8.1(4.4–15.1)	0.014
30-day mortality	13.2%	8.9%	18.1%	<0.001
30-day to 1-year mortality	14.4%	8.5%	22.5%	<0.001

a-eGFR, estimated glomerular filtration rate upon admission (mL/min/1.73 m^2^); PCI, percutaneous coronary intervention; CABG, coronary artery bypass graft; ACEI, angiotensin-converting enzyme inhibitors; ARB, angiotensin II receptor blockers. Continuous variables are presented as median values (with interquartile ranges). Categorical variables are presented as percentages.

* With PCI or CABG.

† With primary PCI or a thrombolytic.

Impaired admission eGFR had no impact on the incidence of AKI, which was similar between the two groups. Patients with an impaired admission eGFR were hospitalized significantly longer and had significantly greater 30-day and 30-day to 1-year mortality rates than those with an admission eGFR≥60 mL/min/1.73 m^2^ (Table 2).

### Comparison of demographic and clinical characteristics based on admission eGFR and the presence of AKI

Among the patients who had an admission eGFR≥60 mL/min/1.73 m^2^, those who had AKI tended to be older (p<0.001), were more likely to have a HR>100 beats/min (p = 0.013) and were more likely to have a history of diabetes (p = 0.009). Among patients with admission eGFRs<60 mL/min/1.73 m^2^, there were no significant differences between subgroups with and without AKI (Table 3).

**Table 3:** Comparison of demographic and clinical characteristics based on admission eGFR and AKI development.

	admission eGFR≥60		admission eGFR<60		
Characteristics	without AKI (n = 387)	with AKI (n = 60)	without AKI (n = 320)	with AKI (n = 61)	p-value
Age (y)	59 (48–70)	70 (55–78)*	69 (61–75)	72 (61–77)	<0.001
Male	73.1%	65.0%	57.8%	57.4%	<0.001
Hypertension	56.6%	66.7%	81.3%	85.2%	<0.001
Current smoker	45.7%	36.7%	26.9%	31.1%	<0.001
Diabetes	17.3%	31.7%†	32.2%	39.3%	<0.001
Previous PCI	6.2%	10.0%	12.5%	4.9%	0.019
Previous CABG	5.4%	3.3%	12.2%	13.1%	0.003
Prior CAD	10.9%	10.0%	20.9%	21.3%	0.001
Previous AMI	21.0%	13.3%	21.3%	19.7%	<0.009
Prior use of ACEIs/ARBs	29.9%	38.3%	54.7%	50.8%	<0.001
STEMI	55.0%	61.7%	41.3%	55.7%	<0.001
Admission SBP<100 mm Hg	2.8%	8.3%	8.2%	16.4%	<0.001
Admission HR ‡	80 (70–92)	86 (70–100)	80 (70–97)	88 (77–110)	0.047
Admission HR >100 ‡	11.6%	23.3%**	19.4%	26.2	0.002

eGFR, estimated glomerular filtration rate (mL/min/1.73 m^2^); PCI, percutaneous coronary intervention; CABG, coronary artery bypass graft; CAD, coronary artery disease; AMI, acute myocardial infarction; ACEI, angiotensin-converting enzyme inhibitors; ARB, angiotensin II receptor blockers; STEMI, ST elevation myocardial infarction; aSBP, systolic blood pressure; HR, heart rate. Continuous variables are presented as median values (with interquartile ranges) and were analyzed by the Kruskal-Wallis test followed by Dunn's post-test. Categorical variables are presented as percentages and were analyzed by χ^2^ statistics with Bonferroni correction for post-test multiple comparisons.

* p<0.001,

† p = 0.009,

** p = 0.013, admission eGFR≥60, with AKI versus without AKI,

‡ (beats/min).

### Influence of admission eGFR and AKI on mortality—univariate analysis

**Figure 1:**
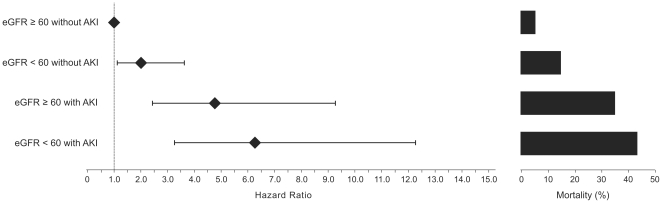
Hospital admission eGFR, AKI development and 30-day mortality rates after acute myocardial infarction. Hazard ratio (Cox multivariate analysis, left) and crude mortality (right).

**Figure 2:**
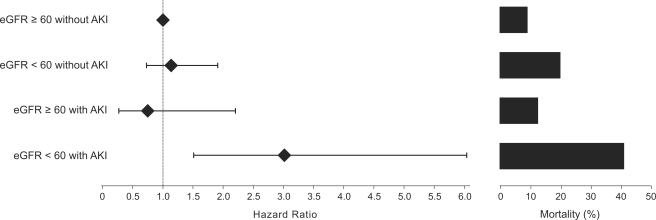
Hospital admission eGFR, AKI development and 30-day to 1-year mortality rates after acute myocardial infarction. Hazard ratio (Cox multivariate analysis, left) and crude mortality (right). Note that only the combination of an admission eGFR<60 mL/min/1.73 m^2^ with AKI was associated with a higher late mortality. * 30-day to 1-year mortality rates were estimated for patients who survived for 30 days after AMI.

Among the patients who did not develop AKI, an impaired admission eGFR was associated with significantly higher 30-day and 30-day to 1-year mortality rates compared to patients with an admission eGFR≥60 mL/min/1.73 m^2^ (figures 1 and 2 and Table 4).

**Table 4:** Influence of impaired admission eGFR on 30-day and 30-day to 1-year mortality rates with and without AKI development (univariate analysis).

	a-eGFR≥60		a-eGFR<60		p-value
Mortality at day 30	n	% (n)	n	% (n)	
Without AKI	387	4.9% (19)	320	13.4% (43)	<0.001
With AKI	60	35% (21)	61	42.6% (26)	0.39

Mortality at 30-days to 1-year*	n	% (n)	n	% (n)	
Without AKI	308	8.1% (25)	217	19.8% (43)	<0.001
With AKI	35	11.4% (4)	32	40.6% (13)	0.006

a-eGFR, admission estimated glomerular filtration rate (mL/min/1.73 m^2^); AKI, acute kidney injury.

* estimated for those surviving at day 30 and who had complete follow-ups for up to one year or death.

Among the patients who developed AKI, the 30-day mortality rate was similar between the groups with preserved or impaired admission eGFR. The 30 day to 1-year mortality rate was significantly higher in patients with an impaired admission eGFR and AKI compared to patients with an admission eGFR≥60 mL/min/1.73 m^2^ and AKI (figures 1 and 2 and Table 4).

### Influence of admission eGFR and AKI on mortality—Cox multivariate analyses

The association between mortality and impaired admission eGFR and/or AKI development was evaluated among the four groups.

#### 30-day survival

**Figure 3:**
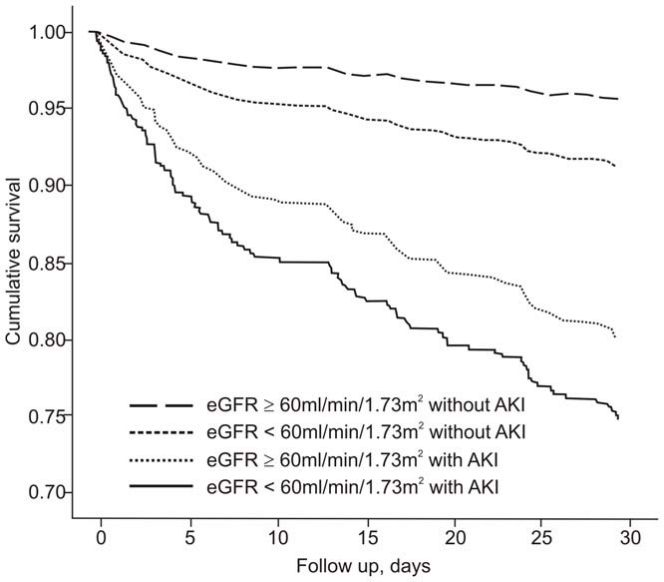
COX curve for 30-day survival among the four groups divided into admission eGFR and AKI development. Admission eGFR, estimated glomerular filtration rate upon admission (mL/min/1.73 m^2^); AKI, acute kidney injury. For the comparison between admission eGFR≥60 without AKI and admission eGFR<60 without AKI, p = 0.020; between admission eGFR≥60 without AKI and admission eGFR≥60 with AKI, p<0.001; for admission eGFR≥60 without AKI and admission eGFR<60 with AKI, p<0.001.

An admission eGFR<60 mL/min/1.73 m^2^ without AKI was associated with an adjusted hazard ratio (AHR) for death of 2.00 (95% CI 1.11–3.61). Patients with a non-impaired admission eGFR and AKI had a death AHR of 4.76 (95% CI 2.45–9.26). The patients with an impaired admission eGFR and AKI had the worst outcome with a mortality AHR of 6.27 (95% CI 3.20–12.29). The reference group was composed of patients with an admission eGFR≥60 mL/min/1.73 m^2^ without AKI (figures 1 and 3 and table 5).

**Table 5:** Cox proportional hazards model for 30-day mortality.

Groups	AHR (95% CI)	p-value
admission eGFR≥60 without AKI (n = 387)	1.0	
admission eGFR<60 without AKI (n = 320)	2.00 (1.11–3.61)	0.020
admission eGFR≥60 with AKI (n = 60)	4.76 (2.45–9.26)	<0.001
admission eGFR<60 with AKI (n = 61)	6.27 (3.20–12.29)	<0.001

eGFR, estimated glomerular filtration rate (mL/min/1.73 m^2^); AKI, acute kidney injury; AHR, adjusted hazard ratio; CI, confidence interval.

The model was adjusted for age creatine phosphokinase-MB, and admission glycemia (categorized by quartiles with the first as a reference), gender (females were used as the reference), history of prior coronary artery bypass graft, ST elevation myocardial infarction, history of diabetes, history of hypertension, admission Killip class >I, systolic blood pressure <100 mmHg, admission heart rate >100 beats/min, clopidogrel use during hospitalization, use of diuretics, coronary angiography during hospitalization, reinfarction, severe systolic left ventricular dysfunction and any percutaneous coronary intervention performed during hospitalization.

#### 30-day to 1-year survival

**Figure 4:**
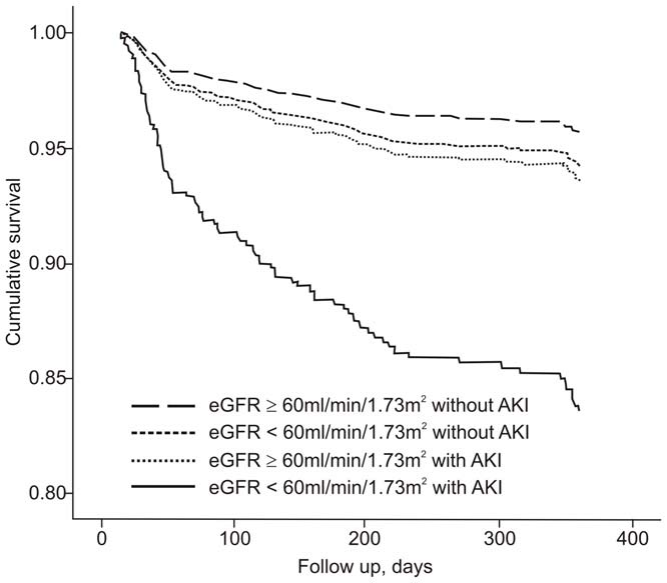
COX curve for 30-day to 1-year survival among the four groups divided into admission eGFR and AKI development. Admission eGFR, estimated glomerular filtration rate upon admission (mL/min/1.73 m^2^); AKI, acute kidney injury. P = 0.002 for the comparison between admission eGFR≥60 without AKI and admission eGFR<60 with AKI, while the differences between the others groups with an admission eGFR≥60 without AKI were non-significant. * 30-day to 1-year mortality rates were estimated for patients who survived for 30 days after AMI.

The 30-day to 1-year survival rate was estimated for those patients who survived for 30 days after AMI. Neither an impaired admission eGFR without AKI nor a non-impaired admission eGFR with AKI were associated with late mortality. In contrast, an impaired admission eGFR with AKI was associated with a death AHR of 3.05 (95% CI 1.50–6.19) (figures 2 and 4 and table 6).

**Table 6:** Cox proportional hazards model for 30-day to 1-year mortality*.

Groups	AHR (95% CI)	p-value
admission eGFR≥60 without AKI (n = 308)	1.0	
admission eGFR<60 without AKI (n = 217)	1.12 (0.65–1.89)	0.696
admission eGFR≥60 with AKI (n = 35)	0.75 (0.26–2.16)	0.588
admission eGFR<60 with AKI (n = 32)	3.05 (1.50–6.19)	0.002

* Estimated for patients who survived for 30 days after AMI.

eGFR, estimated glomerular filtration rate (mL/min/1.73 m^2^); AKI, acute kidney injury; AHR, adjusted hazard ratio; CI, confidence interval.

The model was adjusted for age, admission glycemia (categorized by quartiles with the first as a reference), gender (females were used as the reference), ST elevation myocardial infarction, history of diabetes, history of hypertension, prior use of angiotensin-converting enzyme inhibitors or angiotensin II receptor blockers, admission Killip class >I, admission heart rate >100 beats/min, clopidogrel use during hospitalization, diuretics use, coronary angiography during hospitalization, reinfarction, severe systolic left ventricular dysfunction and any percutaneous coronary intervention performed during hospitalization.

## Discussion

### Influence of admission eGFR on mortality due to AKI

Previous studies have demonstrated that either an impaired renal function at admission or the subsequent development of AKI negatively affects the outcome of patients suffering from an AMI [9], [10], [17], [18]. However, none of the studies prospectively and simultaneously assessed the role of an impaired admission eGFR and AKI development, as defined by RIFLE criteria, on the outcome of AMIs.

In this study, the use of Cox multivariate analysis clearly revealed that the development of AKI was the predominant factor associated with increased 30-day mortality, while an impaired admission eGFR was strikingly associated with an increased long-term mortality in patients with AMI-associated AKI. In fact, during the period from 30 days to one year after hospital admission, only the patients with an impaired admission eGFR and AKI had significantly lower survival rates. These results suggest that early mortality was largely related to the effects of AKI, while long-term outcomes were influenced by AKI development in addition to previously impaired renal function.

The factors leading to the remarkably higher mortality among patients with an impaired admission eGFR and AMI-associated AKI are not evident. One possible explanation for these poorer outcomes may be the combination of a powerful AKI-induced surge of inflammation along with the high prevalence of cardiovascular risk factors, such as older age, diabetes, hypertension and a pro-inflammatory milieu already present in the group of patients with impaired admission eGFR [11], [19]. In addition, we observed differences in the AMI treatment received in the two groups; patients in the group with admission eGFRs<60 mL/min/1.73 m^2^ were less likely to receive pharmacological therapy or to undergo interventional procedures. Other authors have also observed that AMI patients with an impaired admission eGFR were offered less AMI treatment [20]–[23]. More severe cardiac disease was also associated with higher mortality in the current study. However, an association between an impaired admission eGFR and the development of AKI remained independently associated with a higher mortality hazard after controlling for variables possibly related to cardiac function, past history and treatment. Similarly, the overlap between impaired admission eGFR and AKI continued to be independently associated with a higher mortality hazard after correction for the characteristics that distinguish the group with impaired admission eGFRs from the rest of the patient groups.

A limited number of studies have assessed the role of admission eGFR on the outcome of AMI-associated AKI. A study on STEMI patients [5] revealed that AKI, arbitrarily defined as a creatinine elevation >0.5 mg/dL during hospitalization, was associated with increased 30-day and 1-year mortality rates. The study did not find any effect of the admission renal function on mortality due to AKI. It is important to note the differences between this study and our own. We used the RIFLE definition and a time frame of seven days for AKI diagnosis, and we compared the mortality data among the groups by a multivariate Cox proportional hazards regression. On the other hand, Goldberg et al. non-discriminately used an arbitrary absolute SCr increase during any time point of hospitalization for AKI diagnosis and compared the mortality by logistic regression. Another study [24] evaluating AMI patients showed that AKI, defined as a ≥25% decrease in eGFR at any point during hospitalization, was associated with a higher 1-year mortality rate. The authors used univariate analysis to assess the relationship between AKI and chronic kidney disease (CKD), diagnosed as an admission eGFR between 15 and 59 mL/min, and they concluded that AKI was a risk factor for 1-year mortality independent of admission eGFR. This conclusion must be taken with reservations because they did not control for any potential confounding variables in their analysis. Parikh et al. [6] examined the long-term mortality rate after post-MI AKI in a large cohort of patients. In a secondary analysis, the authors suggested that AKI in addition to CKD was associated with a lower risk of death compared to AKI without CKD. The inconsistency of these results with those of the present study could be due to the design differences between the two studies and to the limitations of the study by Parikh et al. [25]. We used RIFLE criteria during the first week of hospitalization to diagnose AKI, while Parikh et al. defined AKI by graded absolute changes in the serum creatinine at any point during hospitalization. As the authors acknowledged in their manuscript, “sicker patients are more likely to have longer hospitalizations and thus undergo more blood work and assessment of renal function, increasing the probability of detecting a change in serum creatinine level”. Another important difference between ours and Parikh's' studies is that we assessed data from 2004 to 2008 in a prospective database, and Parikh et al. used a retrospective database to assess patients hospitalized from 1994 to 1996. As they indicated in their manuscript, the treatment of AMI has substantially improved in the last decade. Further limitations that may affect their secondary analysis are acknowledged by the authors, including the high number of excluded patients (93,784–40% of the total cohort) and the lack of information concerning the pharmacological treatment of these patients, making it impossible to control for these therapies in the Cox analysis, as was done in the present study. Finally, the left ventricular ejection fraction (a strong predictor of long-term survival after AMI) was unavailable in 39.4% of the non-AKI patients in this previous study.

Overall, studies regarding the prognostic effect of pre-existing renal dysfunction on mortality after AKI have reported conflicting results. Some reported a decrease in early mortality in patients with overlapping AKI and CKD compared to the controls in unselected cohorts of critically ill patients [26]–[28]. Conversely, a study by Kolli et al, assessing 1,359 cardiac surgery patients [13] showed that an impaired admission eGFR (<60 mL/min) was associated with a higher mortality rate in patients who developed AKI. Similarly, a study by Wu et al, which analyzed a very large cohort of patients who developed postoperative AKI showed that those patients with previous CKD had a higher risk of long-term mortality than the patients without CKD [16]. Recently, an interesting and elegant experimental study by Skott et al, showed that even mild CKD led to an increased mortality rate in a rodent model of AKI and multiple organ failure [14].

### Influence of admission eGFR on the incidence of AKI

In this study, there was no difference in the incidence of AKI between patients with an admission eGFR<60 mL/min or an eGFR≥60 mL/min. Similarly, Lombardi et al. [29] studied 1,749 patients after cardiac surgery and found no difference in the rates of AKI development (41.9 versus 43.4%, respectively) between patients with an admission eGFR<60 or an eGFR≥60 mL/min. Other studies have reported an association between pre-existing CKD and AKI development [5], [30]. It is unclear whether this association exists as a true primary cause-and-effect relationship or if this association is due to confounding secondary comorbidities that are associated with CKD, the increased exposure of this population to nephrotoxic insults or the study bias. In fact, most of the studies correlating increased AKI incidence with previous CKD utilized a retrospective design with methodological shortcomings, including improper adjustments for comorbidities in multivariate analyses. Furthermore, these studies often lack a standardized protocol for the diagnosis of CKD and AKI.

Although not assessed in the present study, proteinuria, a marker of renal injury, has been recently identified as an important risk factor for the development of AKI in various situations, such as following cardiac surgery [31]–[35].

### Study limitations

This was a single-center observational prospective cohort study.

Systolic left ventricular function as assessed by echocardiography was not always determined by the same observer.

The present study cannot be used to determine the effect of CKD on the incidence and mortality of AKI because the definition of CKD [36] requires the documentation of kidney damage for three or more months. This time assessment is obviously difficult to obtain in this type of study, as most patients have not undergone a renal function evaluation prior to their admission for an acute illness.

AKI incidence was probable underestimated. The reference SCr used for AKI diagnosis was the one obtained at the hospital admission. It is likely that some patients were hospitalized already with AKI, but because there was no SCr increase from the reference SCr during the hospital stay, they were misdiagnosed as non-AKI.

Finally, SCr measurements were performed on a clinical need basis after discharge from the intensive care unit, which could also lead to an underestimation of the incidence of AKI.

### Conclusions

AKI development was negatively associated with the early outcomes of AMI in patients with either non-impaired or impaired admission eGFR. On the other hand, the presence of a low admission eGFR in patients with acute myocardial infarction-associated AKI was clearly associated with a significantly poorer long-term prognosis. Additionally, this study confirmed, using RIFLE criteria that AKI development in AMI patients is frequent and is associated with a high mortality rate.

These results strongly suggest that patients with an impaired admission eGFR should have their renal function monitored closely after an acute myocardial infarction. Clinical measures for the prevention and early diagnosis of AKI must be taken to potentially increase the survival rates of these patients. Moreover, patients with an impaired admission eGFR who develop AKI must have an extremely careful and detailed long-term follow-up to try to avoid the poor long-term outcomes described in this study.

## Methods

### Ethics statement

The study protocol was approved by the Institutional Ethics Committee on September 21, 2009 (“Comitê de Ética em Pesquisa em Seres Humanos da Faculdade de Medicina de Sao José do Rio Preto”, Sao José do Rio Preto, Brazil, process 4988/2009), who agreed that informed consent was unnecessary due to the purely observational and non-interventional nature of this study (“Resolução CNS 196/96”).

### Patients

A total of 1,012 consecutive patients (October 2004–December 2008) with a STEMI or with a NSTEMI [37] were assessed using a prospective database on thoracic pain from a single center.

Among the 1,012 patients, 184 were not included in the study because they met the following exclusion criteria: admission SCr≥6.0 mg/dL, admission eGFR<15.0 mL/min/1.73 m^2^ or chronic dialysis (21 patients); death within 48 hours of admission (49 patients); lack of at least two SCr measurements in the first seven days of hospitalization (94 patients); obstructive AKI (3 patients); and a hospital stay <48 hours (17 patients).

Only the first hospital admission was considered if a patient had more than one hospitalization for AMI during the study period.

### Systolic left ventricular function

Systolic left ventricular function was classified as either normal function or mild, moderate or severe dysfunction and was assessed by echocardiography (based on medical need) in 85.6% of the patients [38].

### SCr measurements, eGFR calculation and diagnostic criteria for AKI

The SCr level was obtained at the time of hospital admission and daily during the intensive care unit (ICU) stay. After ICU discharge, the SCr was measured as needed. The SCr was assessed using the Jaffé colorimetric method (ADVIA™ 1650, Bayer, Germany).

The admission eGFR was assessed by the Modification of Diet in Renal Disease (MDRD) formula [39] (mL/min/1.73 m^2^): GFR = 186.3×SCr ^−1.154^ * age ^−0.203^ * 1.212 (if patient is black) * 0.742 (if female). It should be noted that this equation was not specifically validated for this study population.

Patients were divided into 4 subgroups:

admission eGFR≥60 without AKI

admission eGFR<60 without AKI

admission eGFR≥60 with AKI

admission eGFR<60 with AKI

Patients were diagnosed with AKI if they had a SCr increase of ≥1.5-fold over the admission value within the first seven days of hospitalization (RIFLE criteria, stage Risk). The RIFLE criteria definition of AKI using urinary output (<0.5 mL/kg for 6 h for Risk) was not used in this study [40].

### Outcomes

The primary endpoints were death from any cause within 30 days and 30-day to 1-year mortality for those patients who survived after 30 days. Patient follow-up after discharge was conducted either by an electronic hospital system records review or by mail or telephone contact of patients.

### Statistical Analysis

Patients were categorized based on their admission eGFR (<60 or ≥60 mL/min/1.73 m^2^) and on whether they developed AKI during their hospitalization. The demographics and clinical characteristics (presented as median values with interquartile ranges) were compared by the Student's t-test, Mann-Whitney test or Kruskal-Wallis test followed by Dunn's post-test for continuous variables. The categorical variables (presented as numbers and percentages) were compared using either χ^2^ statistics or Fisher's exact test. Bonferroni corrections were used for post-test multiple comparisons.

Separate analyses were performed on the 30-day mortality rates after the onset of AMI (n = 828) and on the 30-day to 1-year mortality rates for those patients (n = 699) who survived past day 30. Patients who had been part of the follow-up period for less than one year were not included (n = 107) in the descriptive and univariate analyses of the mortality rates at that time point.

We performed multivariate Cox proportional hazards regression analyses to evaluate the two mortality periods. The association between mortality and admission eGFR and AKI development in the four subgroups in which patients were divided was evaluated, controlling for clinically important variables and for variables with a p-value<0.15 in the univariate analyses of mortality in each period. The proportional hazards test and the plotted cumulative survival estimate after the ln (-ln) transformation suggested that the hazards of these variables were proportional for the period analyzed.

In the first period of multivariate Cox proportional hazards regression analysis, the follow-up was censored at day-30 and was adjusted for age, creatine phosphokinase MB (CPK-MB) and admission glycemia (categorized by quartiles with the first as a reference), gender (females were used as the reference), history of prior CABG, STEMI, history of diabetes, history of hypertension, admission Killip class >I, SBP<100 mmHg, admission HR >100 beat/min, clopidogrel use during the hospitalization, use of diuretics, coronary angiography during the hospitalization, reinfarction, severe systolic left ventricular dysfunction (LVD) and any PCI performed during the hospitalization.

The multivariate Cox proportional hazards regression analysis of 30-day to 1-year mortality rates included all patients who survived for 30 days after AMI with censoring at 365 days. The controlling variables were the same used for the 30-day analysis with the exception of CPK-MB, history of prior CABG, SBP<100 mmHg and reinfarction. The prior use of ACEIs or ARBs was also controlled in this analysis.

Differences were considered statistically significant by a two-tailed p-value<0.05 and a confidence interval (CI) of 95%.

Analyses were performed with SPSS statistical software (version 15.0, Chicago, IL, USA).
